# Supplementary material for: Nonlinear photon-atom coupling with 4Pi microscopy
Source: Nat Commun. 2017 Oct 31;8:1200. doi: 10.1038/s41467-017-01495-3 (PMC5663764; doi:10.1038/s41467-017-01495-3)
Supplement: Supplementary file 1 — Supplementary Information [file 41467_2017_1495_MOESM1_ESM.pdf]

Supplementary Note 1: Optical setup

The Gaussian probe beam is delivered from a single mode fibre, collimated and split into two paths (Supplementary Figure 1). The power ratio in the two paths is controlled by a half-wave plate and a polarizing beam splitter. Half- and quarter-wave plates ensure the same polarization (σ^-) in both paths at the position of the atom. After passing through the lens pair, the probe light is coupled into single mode fibres connected to avalanche photodetectors. We optimize the fibre couplings to collect the probe light and measure 40% coupling loss that is due to imperfect mode matching.

We trap single ^{87}Rb atoms with a red-detuned far-off-resonant dipole trap (FORT) at 851 nm. The circularly polarized (σ^+) beam is focused to a waist $w_0 \approx 1.4\,\mu$m, which results in a trap depth of $U_0 = k_B \times 1.88$ mK. The position of the trap is adjusted to maximize the collected atomic fluorescence at the detectors D_1 and D_2. In addition, we use a blue-detuned FORT at 761 nm in standing wave configuration overlapping with the red-detuned FORT to increase the axial confinement. The blue-detuned FORT is linearly polarized and has a trap depth of approximately 0.1 mK along the optical axis.

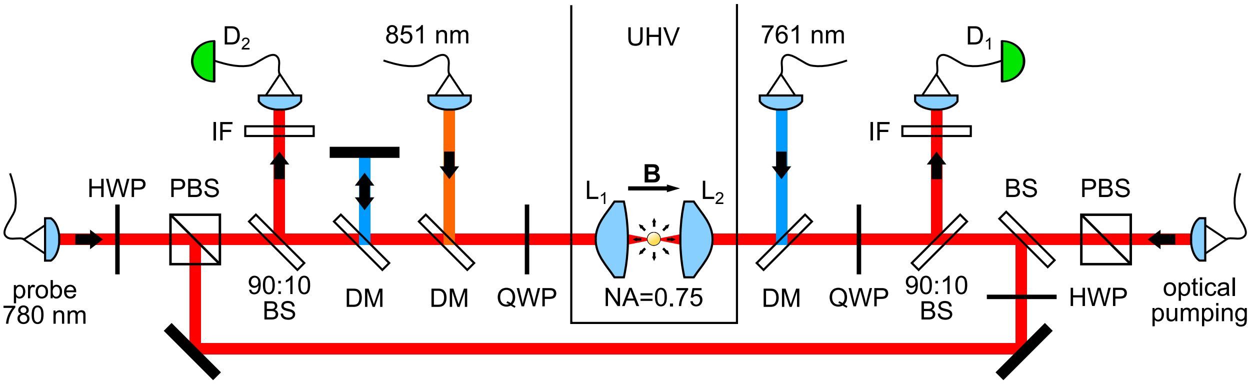

Supplementary Figure 1: **Optical setup.** D_1, D_2: avalanche photodetectors (APDs), IF: interference filter, HWP, QWP: half- and quarter-wave plates, (P)BS: (polarizing) beam splitter, DM: dichroic mirror, L_1, L_2: high numerical aperture lenses, **B**: magnetic field, UHV: ultra-high vacuum chamber.

Supplementary Note 2: Experimental sequence and postselection of atom position

To fully utilize the 4Pi arrangement the atom needs to placed at an anti-node of the probe field. Unfortunately, the interference pattern of the probe field changes over time owing to slow drifts in the optical path lengths. The probe-atom coupling is further affected by similar drifts of the optical lattice, and the probabilistic loading into particular lattice sites. Here we exploit that once an atom is loaded, the timescale for a transmission experiment is much shorter (milliseconds) than the timescale of the drifts (minutes). Therefore, each experimental cycle consists of two independent transmission experiments: one to check whether the atom is trapped at the right position and one to determine the light-atom interaction. In the actual sequence we first perform the light-atom interaction experiment before checking the atom position. In this way we minimize the effect of recoil heating from the probe field.

The experiment begins upon the loading of a single atom. We then perform polarization gradient cooling for 5 ms (Supplementary Figure 2), which cools the atom to a temperature of about 16 µK. A bias magnetic field of 0.74 mT is applied along the optical axis, and the atom is prepared in the $5S_{1/2}$, F=2, m_F=-2 state by optical pumping. Next, two probe fields are applied each for 1 ms, separated by a 4 µs pause. We tune the frequency of the first probe, for example, to obtain the transmission spectra shown in Fig. 2 of the main article. The second probe cycle is used to check whether the atom has been trapped at an anti-node of the probe field. For this, the frequency of the probe field is set to be resonant with the atomic transition. Subsequently, we perform a reference measurement to obtain the instantaneous probe power. We first optically pump the atom to the $5S_{1/2}$, F=1 hyperfine state, shifting the atom out of resonance with the probe field by 6.8 GHz, after which we reapply the two probe fields. The detection events at avalanche photodetectors D_1 and D_2 are recorded during all probe cycles.

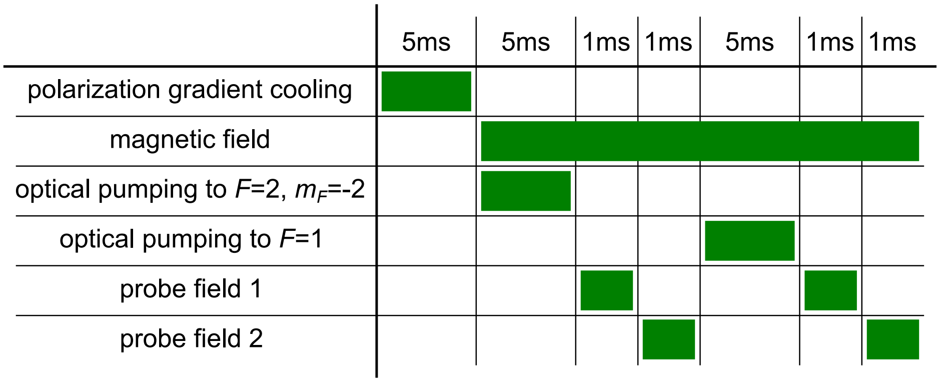

Supplementary Figure 2: **Experimental sequence.**

We illustrate the postselection procedure for the case in which the probe field during the first probe cycle is resonant with the atomic transition. Supplementary Figure 3a,b shows the histogram of detected photons in the first and second probe cycle, respectively. The position of the atom is postselected based on the detected transmission during the second probe cycle. For an atom loaded into a desired site of the potential well, the transmission is low. Hence, we discard detection events in the first probe cycle if the number of photons detected in the second cycle is above a threshold value. Supplementary Figure 3c shows the histogram of detected photons in the first probe cycle after postselection. For the transmission measurements shown in Fig. 2 and Fig. 3 of the main article, we use a photocount threshold that selects approximately 0.5% of the total events, trading off between data acquisition rate and selectiveness of the atomic position. For the case of one-sided illumination, this postselection procedure does not change the observed

transmission (Supplementary Figure 4). In the second-order correlation measurement, we use a higher threshold value to speed up the data acquisition, selecting 10% of the total events. The correlations shown in Fig. 4 of the main article are the result of approximately 200 hours of measurement time.

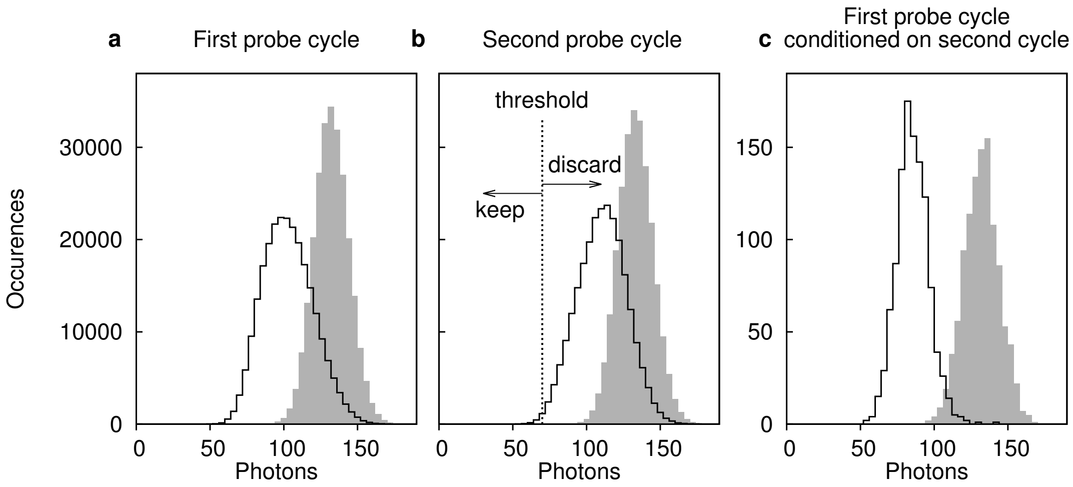

Supplementary Figure 3: **Postselection of atom position.** Photon counting histogram recorded during probe (solid line) and reference (grey) cycle. The total number of detected photons is computed as the sum of detectors D_1 and D_2. **a**, First probe cycle for the case when the probe field is resonant to the atomic transition. **b**, Second probe cycle. The dotted line marks the set threshold for a postselection of approximately 0.5% of the total events. **c**, Resultant events of the first probe cycle conditioned on the second cycle using the marked threshold in **b**.

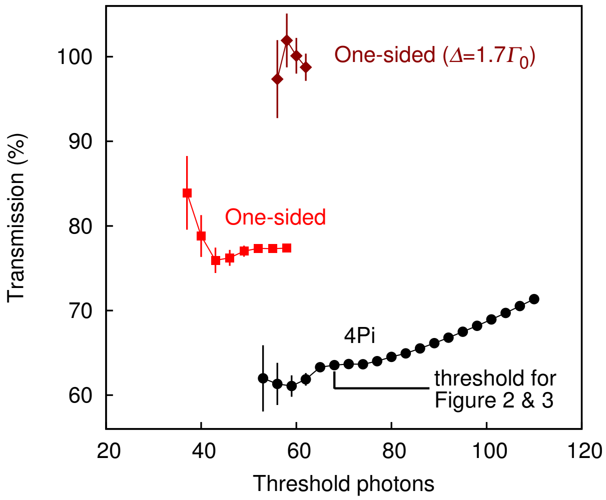

Supplementary Figure 4: **Effect of the postselection procedure on the transmission for 4Pi and one-sided illumination.** Resonant transmission as a function of postselection threshold value for 4Pi illumination (black circles) and one-sided illumination (red squares). The transmission values for one-sided illumination at a detuning $\Delta = 1.7\,\Gamma_0$ are also shown (dark red diamonds). The marked point indicates the threshold value that is used in Fig. 2 and Fig. 3 of the main article. Error bars represent 1 s.d. of propagated Poissonian counting uncertainties.

Supplementary Note 3: Photon statistics of transmitted light

We compute the second order correlation function from the time-tagged photodetection events at detector D_1 and D_2. We sort the photodetection events into a time delay histogram and obtain the normalized correlation function by dividing the number of occurrences by $r_1 r_2 \Delta t T$, where $r_{1(2)}$ is the mean count rate at detector $D_{1(2)}$, Δt is the time bin width and T is the total measurement time. To make the normalization robust against intensity drifts of the probe power and cycle-to-cycle variations of the light-atom coupling, we perform the normalization for every 1 ms-long measurement cycle, obtaining the normalized correlation function $g_i^{(2)}(\tau)$ (index i describes the measurement cycle) and then $g^{(2)}(\tau)$ from the weighted mean

$$g^{(2)}(\tau) = \frac{\sum_{i=1}^{N} g_i^{(2)}(\tau)(r_{1,i} + r_{2,i})}{\sum_{i=1}^{N}(r_{1,i} + r_{2,i})}. \tag{1}$$

Supplementary Figure 5a,b shows $g^{(2)}(\tau)$ around $\tau = 0$ and for longer time delays. For large τ, the correlation disappears, and $g^{(2)}(\tau)$ approaches unity. However, for $100\,\text{ns} < \tau < 1\,\mu\text{s}$, $g^{(2)}(\tau)$ shows super-Poissonian intensity correlations $g^{(2)}(\tau) > 1$. Similar correlations have been observed in the fluorescence of single atoms in dipole traps induced by the atomic motion through the trap [1, 2].

Although the amplitude of the correlations is small, we nevertheless perform a deconvolution for a better comparison to Eq. 2 of the main article. For diffusive motion the correlations are expected to decay exponentially, thus we fit $f(\tau) = 1 + a_0 \exp(-\tau/\tau_d)$ to $g^{(2)}(\tau)$, resulting in amplitude $a_0 = 0.019(2)$, decay time constant $\tau_d = 0.71(8)\,\mu\text{s}$, with a reduced $\chi^2 = 1.07$ (Supplementary Figure 5b, black solid line). We note that the timescale τ_d of these correlations is much larger than the excited state lifetime $1/\Gamma_0 = 26.2\,\text{ns}$. Figure 4 of the main text shows the second order correlation function corrected for the diffusive motion, that is, after division by $f(\tau)$. No additional correlations are present in the transmitted light during the reference cycle in which the atom is not resonant with probe field (Supplementary Figure 5c).

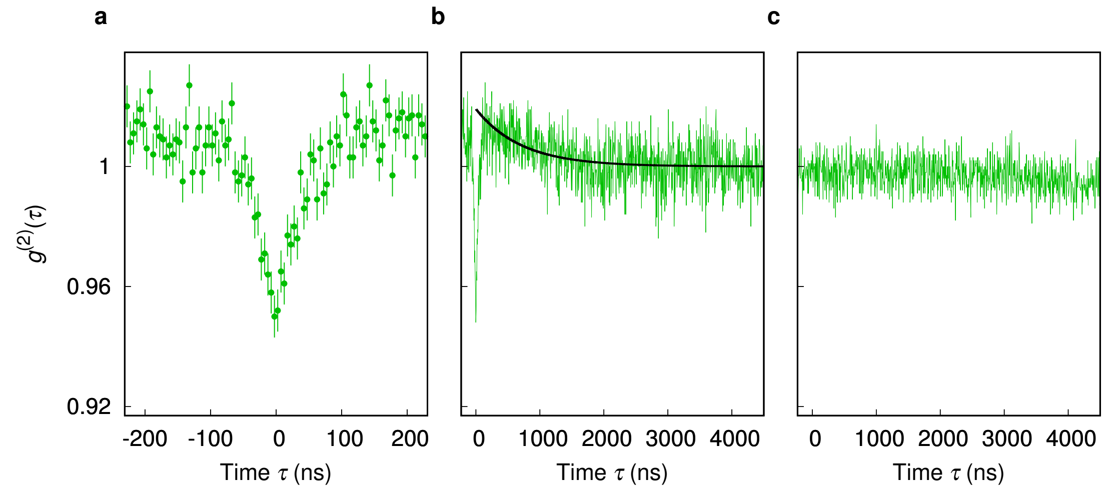

Supplementary Figure 5: **Photon bunching due to atomic motion.** **a,** Normalized second order correlation function without deconvolution of the diffusive atomic motion. **b,** Same as **a** but with extended range. Solid line is a fit to $f(\tau) = 1 + a_0 \exp(-\tau/\tau_d)$ with $a_0 = 0.019(2)$, $\tau_d = 0.71(8)\,\mu\text{s}$ and a reduced $\chi^2 = 1.07$. **c,** Same as **b** but computed from events during the reference cycle in which the atom is not resonant with probe field. Error bars represent 1 s.d. of propagated Poissonian counting uncertainties.

Supplementary References

---

[1] Gomer, V. *et al.* Decoding the dynamics of a single trapped atom from photon correlations. *Applied Physics B* **67**, 689–697 (1998).

[2] Weber, M., Volz, J., Saucke, K., Kurtsiefer, C. & Weinfurter, H. Analysis of a single-atom dipole trap. *Phys. Rev. A* **73**, 043406 (2006).
